# Mapping Systematic Reviews on Forensic Psychiatric Care: A Systematic Review Identifying Knowledge Gaps

**DOI:** 10.3389/fpsyt.2018.00452

**Published:** 2018-09-25

**Authors:** Katarina Howner, Peter Andiné, Göran Bertilsson, Monica Hultcrantz, Eva Lindström, Frida Mowafi, Alexandra Snellman, Björn Hofvander

**Affiliations:** ^1^Department of Clinical Neuroscience, Centre of Psychiatry Research, Karolinska Institutet, Stockholm, Sweden; ^2^Division for Forensic Psychiatry in Stockholm, Department for Forensic Psychiatry, National Board of Forensic Medicine, Stockholm, Sweden; ^3^Division for Forensic Psychiatry in Gothenburg, Department for Forensic Psychiatry, National Board of Forensic Medicine, Gothenburg, Sweden; ^4^Department of Psychiatry and Neurochemistry, Centre for Ethics, Law and Mental Health, Institute of Neuroscience and Physiology, The Sahlgrenska Academy at University of Gothenburg, Gothenburg, Sweden; ^5^Forensic Psychiatric Clinic, Sahlgrenska University Hospital, Gothenburg, Sweden; ^6^Swedish Agency for Health Technology Assessment and Assessment of Social Services (SBU), Stockholm, Sweden; ^7^Department of Neuroscience, Psychiatry, Uppsala University, Uppsala, Sweden; ^8^Department of Clinical Sciences, Lund, Child and Adolescent Psychiatry, Lund University, Lund, Sweden

**Keywords:** forensic psychiatric care, mentally disordered offenders, risk assessments, pharmacological treatment, psychological interventions, psychosocial interventions, restraint interventions, systematic review

## Abstract

**Background:** Forensic psychiatric care treats mentally disordered offenders who suffer mainly from psychotic disorders, although comorbidities such as personality disorders, neurodevelopmental disorders, and substance abuse are common. A large proportion of these patients have committed violent crimes. Their care is involuntary, and their caregivers' mission is complex: not only to rehabilitate the patient, but also to consider their risk for reoffending and their risk to society. The objective of this overview of systematic reviews is to identify, appraise, and summarize the existing knowledge in forensic psychiatric care and identify knowledge gaps that require further research.

**Methods:** We undertook a systematic literature search for systematic reviews in five defined domains considered important in daily clinical practice within the forensic psychiatric care: (1) diagnostic assessment and risk assessments; (2) pharmacological treatment; (3) psychological interventions; (4) psychosocial interventions, rehabilitation, and habilitation; and (5) restraint interventions. The target population was mentally disordered offenders (forensic psychiatric patients aged >15 years). Each abstract and full text review was assessed by two of the authors. Relevant reviews then were assessed for bias, and those with moderate or low risk of bias were included.

**Results:** Of 38 systematic reviews meeting the inclusion criteria, only four had a moderate risk of bias. Two aimed to incorporate as many aspects of forensic psychiatric care as possible, one investigated non-pharmacological interventions to reduce aggression in forensic psychiatric care, and one focused on women with intellectual disabilities in forensic care. However, most of the primary studies included in these reviews had high risks of bias, and therefore, no conclusions could be drawn. All of our identified domains must be considered knowledge gaps.

**Conclusion:** We could not answer any of our research questions within the five domains because of the high risk of bias in the primary studies in the included systematic reviews. There is an urgent need for more research on forensic psychiatric care since all of our studied domains were considered knowledge gaps.

## Introduction

Mentally disordered offenders in most developed countries are treated according to special regulations in the legal system. Most often, the concept of accountability is applied, and if an offender is found to be unaccountable before a verdict has been reached, he or she will be moved out of the criminal justice system and into a compulsory psychiatric care system (1). These offenders are typically treated in secure or forensic psychiatric hospitals, sometimes alongside prisoners who cannot be managed by prison medical services or psychiatric patients who cannot be managed in general wards. The number of forensic psychiatric beds has increased considerably in many high-income countries (2, 3) and forensic psychiatry often claims a large share of the overall psychiatric budget, while serving a very small share of the psychiatric patient population (4).

Most forensic psychiatric patients suffer from disorders with psychotic symptomatology (5, 6), but comorbidities are very common, especially personality disorders, neurodevelopmental disorders, and substance-related disorders (6). Forensic patients are often marginalized with lack of education and unemployment. Their motivation for treatment can also fluctuate since they often lack insight into their illness and have been admitted involuntarily (7). As opposed to general psychiatric patients, the forensic patient is not only a patient but also an offender. This means psychiatric caregivers must also consider these patients' risks of reoffending and society's need to be protected from violent offenders. Constant risk assessments are necessary during forensic psychiatric treatment, and patients' risk of reoffending contributes to their considerable lengths of stay in forensic psychiatry (5). Rehabilitation, adjustment to society, and reducing the risk for re-offense are all important goals of the forensic psychiatric care system.

In 2016 the Swedish government gave the Swedish Agency for Health Technology Assessment and Assessment of Social Services (SBU) the task of identifying knowledge gaps in the field of forensic psychiatric care. Knowledge gaps were identified when systematic reviews revealed uncertainty about the effect of a specific treatment, or if no systematic review of that treatment was available.

### Objectives

To identify, appraise, and summarize existing knowledge and to identify knowledge gaps in clinically relevant domains of forensic psychiatric care.

### Research questions

Four clinical experts (KH, EL, PA, BH) identified the most relevant clinical domains in forensic psychiatric care. This was done in several meetings when the expert discussed different aspects of the forensic psychiatric care. All four experts have experience from clinical forensic psychiatric care. In addition major patient organizations and nine of the largest forensic psychiatric hospitals in Sweden were also asked to list five areas they wished to prioritize. The following five domains and research questions were selected:

1. Diagnostic assessment and risk assessment in forensic psychiatric care
  Forensic patients spend many years in hospital and their diagnoses should be reassessed over this time. What is the long-term stability of diagnoses in forensic psychiatry? How do regular assessment instruments work in a forensic clinical setting? What would it mean to the patient if a diagnosis were redefined, and how would that affect their treatment? Risk assessments are done regularly in the forensic psychiatric care system, but do we know how these assessments affect outcomes or what instruments should be used?
2. Pharmacological treatment
  In forensic psychiatric care, almost all patients receive pharmacological treatment, which often includes combinations of agents such as antipsychotics, mood stabilizers, sedatives, anxiolytics, and antidepressants, sometimes the administered in higher doses than in general psychiatry. What are the effects and side effects of this pharmacological treatment?
3. Psychological interventions
  Many forensic psychiatric patients receive psychological treatment such as Cognitive Behavioral Therapy (CBT) individually or in group. Do these interventions affect their length of stay, risk of reoffending, and/or risk of relapse in substance abuse?
4. Psychosocial interventions, rehabilitation, and habilitation
  Different kinds of individualized interventions in forensic psychiatric care are administered by occupational therapists, physiotherapists, social workers, and other health care professionals to inpatients as well as outpatients. What do we know about the outcomes of these interventions?
5. Restraint interventions
  All patients in the forensic psychiatric care system are treated involuntarily and can therefore be subject to forced medication, medical restraint, and seclusion. These interventions are often ethically challenging and can be difficult not only for the patients but also for the staff. How do patients experience restraint interventions, and how do they affect treatment and compliance in forensic psychiatry?

## Methods

### Study design

This is an overview of systematic reviews published in peer-reviewed journals. Systematic reviews based on quantitative studies and written in English, Swedish, Norwegian, or Danish were included.

### Population, interventions, control, outcomes (PICO)

The following PICO criteria were used in the literature search:

- Population: offenders aged over 15 years with a severe mental disorder, treated involuntarily in a forensic psychiatric or secure hospital.
- Interventions: interventions in any of the identified domains in forensic psychiatric care (diagnostic assessment and risk assessment; pharmacological treatment; psychological interventions; psychosocial interventions, rehabilitation, and habilitation; and restraint interventions).
- Control: no limitation.
- Outcomes: clinical (symptoms and side effects), re-offense(s), adherence to treatment, social functioning, occupational functioning, cognitive functioning, quality of life, rehospitalization, and accuracy of diagnostic instruments and risk assessments.

### Search strategy

The original literature search was made on October 27, 2016, in 10 different databases: CINAHL, Cochrane Library, Psych Info, Pub Med, Soc Index, Embase, Joanna Briggs Institute Database, Medline, Psychology and Behavioral Science Collection, and Scopus. An updated literature search was made on April 18, 2018, using the same search strategy in all the same databases except the Joanna Briggs. The search strategy is described with examples in Table 1. All search strategies are available in the Supplementary Material.

**Table 1:** Example of literature search strategy.

**Search terms**		**Items found**
**SETTING: PERSONS WITHIN FORENSIC INSTITUTIONS/MENTALLY ILL OFFENDERS**		
1.	(“Forensic psychiatr^*^”[tiab] OR “forensic institute^*^”[tiab] OR “forensic inpatient^*^”[tiab] OR “forensic patient^*^”[tiab] OR “forensic out-patient^*^”[tiab] OR “forensic outpatient^*^”[tiab] OR “forensic clinical practice^*^”[tiab] OR “forensic hospital^*^”[tiab] OR “forensic treatment^*^”[tiab] OR “forensic service^*^”[tiab] OR “forensic ward^*^”[tiab] OR “forensic mental”[tiab] OR “forensic facili^*^”[tiab] OR “forensic clinic^*^”[tiab] OR “forensic neuropsych^*^”[tiab] OR “forensic center^*^”[tiab] OR “forensic unit^*^”[tiab] OR “Forensic setting”[tiab] OR “forensic settings”[tiab] OR “forensic population”[tiab] OR “forensic populations”[tiab] OR “secure psychiatr^*^”[tiab] OR “secure setting^*^”[tiab] OR “secure hospital”[tiab] OR “Maximum secur^*^”[tiab] OR “high secur^*^”[tiab] OR “medium secur^*^”[tiab] OR “low^*^ secur^*^”[tiab] OR “minimum secur^*^”[tiab] OR “forensic^*^ secur^*^”[tiab] OR “secur^*^ forensic^*^”[tiab]) NOT Medline[SB]	159
2.	((Offender^*^[tiab] OR criminal^*^[tiab]OR offending[tiab] OR offend^*^[tiab] OR forensic[tiab] OR incarcerate^*^[tiab] OR justice^*^[tiab] OR delinquent^*^[tiab] OR inmate^*^[tiab] OR correctional[tiab] OR prison^*^[tiab] OR “violent offense^*^”[tiab] OR reoffend^*^[tiab] OR re-offend^*^[tiab]) AND (psychiatri^*^[tiab] OR psycholog[tiab] OR mental^*^[tiab] OR intellectual^*^[tiab] OR Schizo^*^[tiab] OR “personality disorder^*^”[tiab] OR borderline[tiab] OR antisocial[tiab] OR Firesetting^*^[tiab] OR Pyroman^*^[tiab] OR arson^*^[tiab] OR Paraphil^*^[tiab] OR pedophil^*^[tiab] OR paedophil^*^[tiab] OR Hallucinat^*^[tiab] OR “dual disord^*^”[tiab])) NOT medline[SB]	1714
3.	1 OR 2	1791
**COMBINED SETS, LIMITED TO STUDY TYPE: SYSTEMATIC REVIEW**		
4.	3 AND Systematic[SB]	68

*PubMed via NLM 28 October 2016*.

*Title: forensic psychiatry/mentally ill offenders (complimentary search to find non-indexed references)*.

[MeSH], Term from the Medline controlled vocabulary, including terms found below this term in the MeSH hierarchy; [MeSH:NoExp], Does not include terms found below this term in the MeSH hierarchy; [MAJR], MeSH Major Topic; [TIAB], Title or abstract; [TI], Title; [AU], Author; [TW], Text Word; Systematic[SB], Filter for retrieving systematic reviews; ^*^, Truncation.

### Data sources, studies sections, and data extraction

Abstracts identified according to the inclusion criteria were each examined pairwise by four of the authors (KH & BH and PA & EL). If at least one author found an abstract potentially relevant, the full text review was studied. Full text reviews were assessed according to inclusion criteria and most of them did not meet the inclusion criteria and were therefore excluded.

### Data analysis

The quality of the included reviews was assessed independently by three authors (AS, FM, MH). In unclear cases the final decision to exclude a study was made by consensus of the whole group of authors. The quality assessment was made using the AMSTAR checklist^1^ (8, 9), which focuses on how the review was conducted. A good quality review should have an a priori design and a comprehensive literature search and must have assessed and documented the scientific quality of the included studies. The scientific quality of the primary studies in a systematic review, commonly referred to as its risk of bias, reflects the risk that the study results were skewed by weaknesses in the research process. We used a conservative approach; if a feature was not reported, we assumed it was absent.

## Results

**Figure 1:**
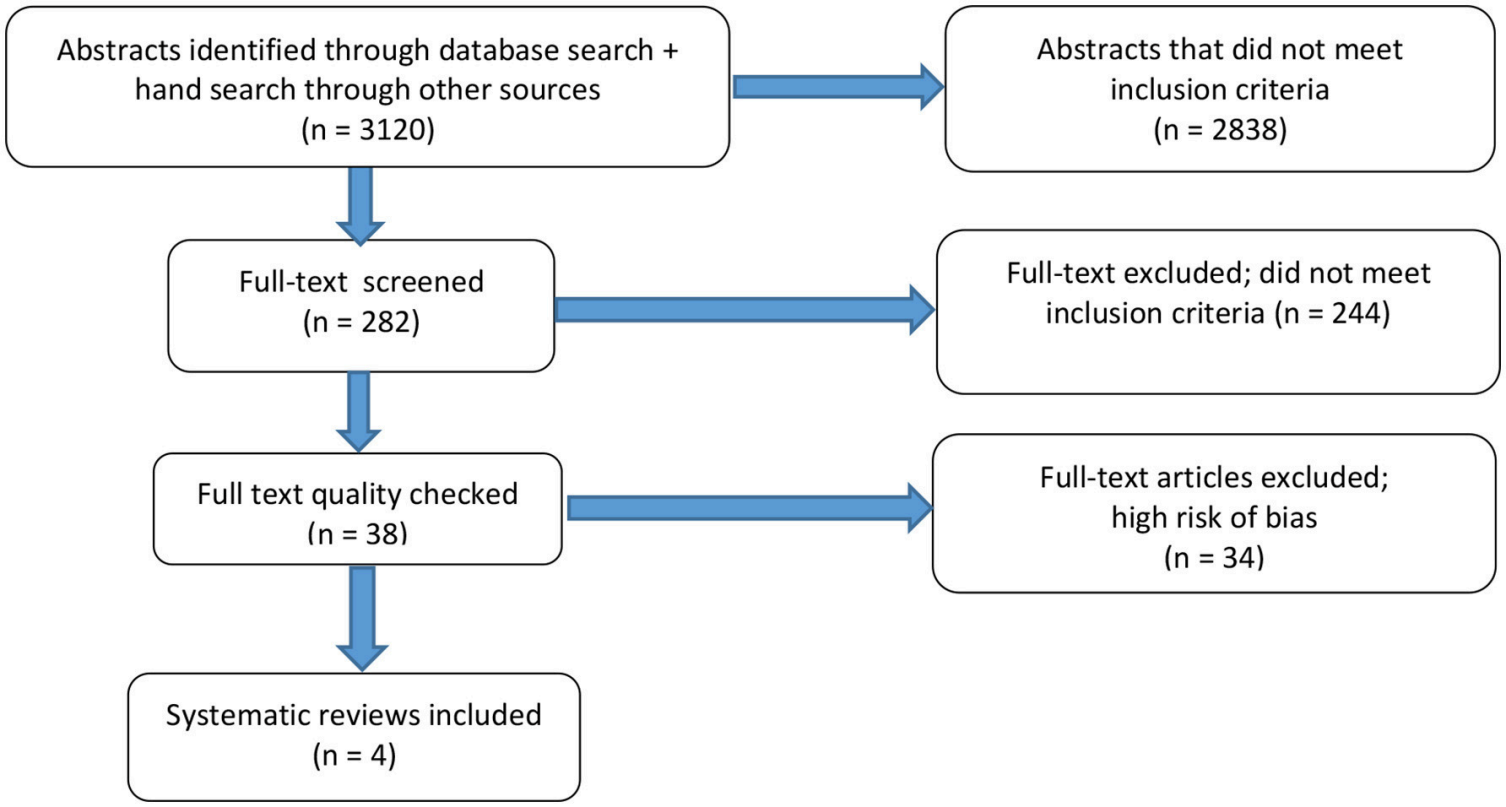
Flowchart of abstracts and articles retrieved from the literature search.

The numbers of abstracts retrieved and articles included and excluded at each stage of the search are presented in a flowchart (Figure 1).

### Study selection and characteristics

Of 38 systematic reviews meeting the inclusion criteria and quality checked using AMSTAR (8, 9), only four were judged to have a moderate risk of bias and were included in this review. The remaining 34 articles were judged to have high risk of bias and were therefore excluded. Two of the included reviews aimed to describe a broad spectrum of interventions used in forensic psychiatric care (10, 11). Another focused on cognitive treatment of female offenders with intellectual disabilities (12), and the fourth study focused on non-pharmacological treatment of aggressive behavior in forensic psychiatric care (13).

### Synthesized findings

The four included reviews are presented in Table 2.

**Table 2:** The four included reviews (moderate risk of bias).

**Title, first author, year, country**	**Objectives**	**Interventions outcomes**	**Number of included studies (participants) study design of included studies**	**Main results and the estimated level of evidence according to the systematic review**
Interventions for adult offenders with serious mental illness—comparative effectiveness review Fontanarosa 2013 USA	To comprehensively review the evidence for treatments for offenders with serious mental ilness (SMI) (i.e., schizophrenia, schizoaffective disorder, bipolar disorder, or major depression)	*Interventions***:** Pharmacologic therapy with clozapine, risperidone, or chlorpromazine, psychological therapies, comprehensive interventions for individuals with a dual diagnosis, high-fidelity integrated dual disorder treatment (IDDT), The Mentally Ill Offender Community Transition Program, Discharge planning interventions that included assistance applying for mental health benefits, interventions coordinated and/or administered by specially trained forensic providers, Interpersonal therapy (IPT) *Outcomes:* suicide and suicide attempts, quality of life, independent functioning, psychiatric symptoms, new mental health diagnosis, substance or alcohol use, hospitalization for SMI, time to re-hospitalization, time to relapse, dangerousness to others, and recidivism and other criminal justice outcomes	Studies = 16 in 19 publications, (*n* = 858) Randomized controlled trials (RCT) and non-randomized (prospective or retrospective) comparative trials Studies must have either randomly assigned patients or facilities to treatments or used an analytic method to address selection bias, such as baseline matching on multiple characteristics, propensity scoring, or other analytic approach	Because of the nature of the available evidence, there was a qualitative synthesis performed: In summary, in an incarceration setting, treatment with antipsychotics other than clozapine appears to improve psychiatric symptoms more than treatment with clozapine. However, this conclusion is based on two trials that poorly described both the treatment and its comparator Likewise, discharge planning with benefit-application assistance appears to increase mental health service use for incarcerated individuals with SMI preparing to re-enter the community. Again, this conclusion is based on only two trials, and whether increased service use will lead to improved patient outcomes remains unclear IDDT also appears to be a promising intervention for reducing psychiatric hospitalization in offenders returning to the community
Psychological interventions for women with intellectual disabilities and forensic care needs: a systematic review of the literature Hellenbach 2015 UK	To examine evidence on psycho-social therapies for the female intellectually disabled population within healthcare forensic facilities	*Interventions***:** Cognitive Behavior Therapy (CBT) *Outcomes*: the existing body of research in relation to evidence-based treatment for women with a diagnosis of ID and mental illness that have forensic care needs	Studies = 4, (*n* = 41, intellectually disabled women) Non-randomized, non-comparative studies	In total, 4 studies were identified that met the inclusion criteria. A range of CBT-orientated group interventions for people with learning disabilities were evaluated, and in most studies improvements were reported in relation to reducing problem behavior. Evidence that has been generated by the studies is, however, limited in its explanatory value because of study design and related methodological issues
Non-pharmacological interventions for reducing aggression and violence in serious mental illness: A systematic review and narrative synthesis Rampling 2016 UK and Italy	To aggregate evidence for non-pharmacological interventions in reducing violence among adults with serious mental illness and personality disorder, and to assess the efficacy of these interventions	*Interventions***:** any form of specific non-pharmacological intervention *Outcomes***:** violence (physical violence, verbal aggression or violent attitudes)	Studies = 23, (*n* = 1,839, a majority studies with mental disordered offenders) Experimental and quasi-experimental study designs that included 7 RCTs	The evidence for non-pharmacological interventions for reducing violence in this population is not conclusive. Long-term outcomes are lacking, and good quality RCTs are required to develop a stronger evidence base
A critical analysis of clinical evidence from high secure forensic inpatient services Tapp 2013 UK	Establish whether services are effective in restoring mental health and reducing risk	*Interventions:* high secure hospital treatment, milieu interventions, environmental, behavioral, psychotherapy, psychoeducation, pharmacological, dietary *Outcomes*: Re-offending, re-admission, mental health, sex offending, social function, aggression, self-harm, institutional behavior/management, iatrogenic effects, quality of life, perceptions of service support and mental health awareness	Studies = 22, (*n* = 2,267, adult detainees in high secure forensic inpatient services) Non-randomized trials. Studies were commonly assessed as being at a potentially high risk of bias from validity threats	There was evidence to indicate that intervention effects differed substantially between studies on the basis of clinical and methodological variability, across participants, comparators, methods, outcomes and quality rating. Therefore, to avoid pooled effects bias and the risk of drawing incorrect conclusions no comparisons were conducted

The results are presented for each domain:

*Domain 1: Diagnostic assessments and risk assessment during forensic psychiatric care*.

We found no systematic review with low or moderate risk of bias focusing on this domain in the targeted population. Systematic reviews of risk assessments have, however, been published in different samples from general psychiatry and prison populations (4, 14).

Domain 2: Pharmacological treatment

Two systematic reviews with moderate risk of bias contained studies focusing on pharmacological treatment: (10, 11). However, the primary studies in the reviews were judged to have a high risk of bias. The primary studies were highly heterogeneous in population, outcomes, methods of measurement, and time frames. Comparisons had been drawn between different antipsychotics, different anticonvulsants, and placebo. The authors of both reviews chose not to perform a meta-analysis of the studies they had included.

Domain 3: Psychological interventions

Four systematic reviews in this domain had a moderate risk of bias (10–13). Again, in all of these, the primary studies were judged to have a high risk of bias. These reviews are helpful in providing information about trends in the data and describing areas where a body of evidence is emerging. The somewhat promising results in this domain, however, should be interpreted with caution because, again, they are based on studies with a high risk of bias. The reviews included studies on CBT and third wave CBT in forensic care for treating aggressive behavior (13), preventing reoffending (10), and interpersonal violence (11), and treating disrupted behavior in women with intellectual disabilities (12).

Domain 4: Psychosocial interventions, rehabilitation, and habilitation

In this domain the three systematic reviews found with a moderate risk of bias (10–12), the included primary studies mainly had a high risk of bias. Hence, these data should be interpreted as providing information about trends and describing areas with an emerging body of evidence. These reviews included studies on therapeutic communities (10, 11), integrated dual disorder treatment (10), and supported housing (13).

We found no systematic reviews in the field of rehabilitation and habilitation.

Domain 5: Restraint interventions

We found no systematic review in this domain.

### Risk of bias

We found no systematic review with a low risk of bias, but four systematic reviews were considered to have a moderate risk. Those four were included in our review, but most of their included primary studies had a high risk of bias; therefore, no quantitative meta-analysis could be performed. The risk of bias in systematic reviews is often the result of high heterogeneity among the included primary studies. Differences were found in many areas including specific interventions, study populations, time frames, and methods of measurement.

## Discussion

### Summary of main findings

The main finding of this study is that very few well-conducted systematic reviews have been published in this area. The four identified systematic reviews were published in 2013–2016, and although new primary studies may have been published since, the reviews we did find indicate a great lack of primary research in the forensic psychiatric setting. In two of our five domains (“diagnostic assessments and risk assessment in forensic psychiatric care” and “restraint interventions”) we found no systematic reviews at all. In the domain of pharmacological treatment we found two systematic reviews, but their primary studies had high risk of bias. The effects and side effects of pharmacological treatment must therefore be considered a knowledge gap. In the domain of psychological interventions we found four systematic reviews, but since most of the included primary studies had high risk of bias, we could not draw any conclusions from them. In the domain of psychosocial interventions, rehabilitation, and habilitation we found three reviews, but all with the same problem as the previous domains: the primary studies had high risk of bias. Based on these results, we can only conclude that all the investigated domains represent important knowledge gaps in forensic psychiatric care.

Because very few studies in forensic psychiatry have low risk of bias, the possibility of conducting systematic reviews that can guide clinical decisions is low. The main reason for the high risk of bias was that several of the included studies lacked randomization and blinding. The broader lack of studies in forensic psychiatry settings may be due to practical problems; recruiting large study populations can also be difficult.

The four systematic reviews we found with moderate risk of bias studied patients in forensic psychiatric care, as defined in the inclusion criteria. Other systematic reviews have used studies in other populations such as prisons or general psychiatry, suggesting that sufficient studies have been conducted in these groups. It is therefore vitally important to perform more research focused on the specific population of offenders with severe mental disorders.

Specific comments on each of the included domains follow.

#### Diagnostic and risk assessments during forensic psychiatric care

The forensic psychiatric population suffers from severe mental disorders, often with extensive comorbidity including substance use disorder, neurodevelopmental disorders, and personality disorders; a large proportion of the population also has a long history of violent behavior (15). In prison samples, many have an antisocial personality disorder and violent behavior, but do not normally suffer from psychotic conditions. Nevertheless, there may be important knowledge and useful information from systematic reviews of studies in prison populations about, for example, the assessment of ADHD (attention deficit/hyperactivity disorder) and substance use disorders. There might also be important findings from reviews in general psychiatry on diagnostic assessments for specific diagnostic groups such as psychotic disorders, bipolar disorders, personality disorders, and neurodevelopmental disorders.

In the forensic psychiatric care system, staff must continually perform risk assessments to support decisions about security measures and learn about patients' risk of reoffending during leaves of absence and discharge. Risk assessments are pivotally important to the clinical practice of forensic psychiatry, and considerable resources are spent on these assessments. Over the last two decades, the practice of risk assessment in Sweden has followed international developments and is conducted as a structured clinical assessment using Swedish versions of instruments such as Historical-Clinical-Risk Management−20 [HCR-20; (16)] and the Psychopathy Check List revised [PCL-R; (17)]. Still there are several unanswered questions in the field of risk assessment. For example, virtually all research has been nomothetic and has thus dealt with numbers of reoffenders in a group rather than to individuals. We still need more research on the ability of these instruments to identify those individuals who reoffend. Such scientific knowledge may then be used for systematic cost–benefit analyses of the use of risk assessments. Systematic reviews of studies in other populations such as in general psychiatry and prisons can provide some information, but it would be preferable to have studies in the specific setting of forensic psychiatry.

#### Pharmacological treatment

Data from the national quality register for Swedish forensic psychiatric care show that patients with psychotic symptoms in forensic psychiatric care are more likely to be treated with a combination of several antipsychotic agents as well as higher proportion of typical antipsychotic agents than patients in general psychiatric care (18). There is also a perception among clinicians in forensic psychiatric care that doses of antipsychotic agents are higher than in general psychiatry. Since treatment-refractory cases are common, and patients often suffer comorbidities such as substance use disorders and personality disorders, their pharmacological treatment can be challenging. The patients may also suffer from somatic disorders, which makes it even more difficult to choose the right agent and adjust dosages. The central position of pharmacological treatment in forensic psychiatric care also makes it particularly important to study side effects. Systematic reviews including prison or general psychiatry populations may contain important knowledge and useful information about the pharmacological treatment of such diagnoses as ADHD, psychotic disorders, bipolar disorders, and personality disorders in combination with substance use disorders. Existing guidelines for the pharmacological treatment of schizophrenia and other psychoses should be considered and may be valid for the forensic group of patients with psychotic disorders. New knowledge is needed about possible differences between psychotic patients in forensic care and those in general psychiatry. An updated systematic review focusing on forensic psychiatric patients is urgently needed.

#### Psychological interventions

Several recommendations in guidelines for treating schizophrenia and other psychotic conditions seem adequate for forensic psychiatric patients, but further studies are needed to evaluate this. Studies are specifically needed to analyze how well these interventions work in a compulsory clinical setting where length of stay is radically different from general psychiatry. Studies are also lacking on how various psychological interventions are affected by different pharmacological regimes and patterns of comorbidity. Systematic reviews of interventions aimed to reduce the risk of reoffending, specific interventions for sexual crimes and intimate partner violence, and the management of violent and antisocial behavior may hold valuable information that could be applicable to the treatment of forensic psychiatry patients.

#### Psychosocial interventions, rehabilitation, and habilitation

We sought knowledge about how we should adjust our patients to a life outside the hospital. The time spent inside the hospital is often very long and how to make a meaningful everyday life inside the ward is challenging. The patients often lack occupation and fulfilled educations. Many of the patients have dropped out of school and are unemployed, and there is a high rate of low literacy among them. Three of the included systematic reviews included studies on therapeutic communities (10, 11), integrated dual disorder treatment (10), and supported housing (13). However, all the included primary studies were assessed high risk of bias.

We found no systematic reviews in the field of rehabilitation and habilitation.

#### Restraint interventions

How restraint interventions are experienced by the forensic psychiatric patient and how they affect different short- and long-term treatment outcomes such as compliance to treatment and reduction of reoffending are of great interest for future investigation. Results from other populations such as general psychiatry could add useful information and knowledge.

### Limitations

Since legislation differs between countries, it is always difficult to compare studies from different settings. However, the international forensic psychiatric population has much in common including psychotic conditions with high comorbidities of substance abuse, personality disorders, and autism spectrum disorders combined with antisocial and violent behavior. As always, when a mapping survey is conducted there are systematic reviews that do meet some, but not all, inclusion criteria or quality demands, meaning that potentially important, and interesting published studies many not been included in reviews or in this overview.

## Conclusions

This overview of systematic reviews provides a systematic description of research activity in practice-relevant fields of forensic psychiatry. Our conclusion is that all our studied domains represent knowledge gaps, and there is an urgent need for more primary studies and systematic reviews in the field of forensic psychiatric care.

### Future directions

The lack of scientific evidence in a field can be described as a knowledge gap. This is not to say that interventions currently used in forensic psychiatry are non-scientific. It does, however, imply scientific uncertainty about treatment effects and side effects and the need for further research in this area. In the absence of scientific evidence for alternative methods, one should adhere to established treatments. Although legislation differs, the involuntary nature of forensic psychiatric care forced upon patients is similar in most countries. Because it is not possible to wait for more research and evidence, considering that the patients need treatment now, one should use the best available evidence. This means relying on available guidelines from general psychiatry as well as using well-tried experience while waiting for new studies and evidence. In Sweden, the SBU has initiated two systematic reviews: one studying the effects and side effects of pharmacological treatment used in forensic psychiatric care, and one focusing on psychological and psychosocial interventions in forensic psychiatric care.

## Author contributions

KH, PA, EL, and BH defined domains, assessed the relevance of abstracts and full text articles, analyzed and interpreted the results, and wrote the manuscript. AS, FM, and MH conducted quality assessments with AMSTAR, extracted data, analyzed and interpreted results, and participated in writing the manuscript. GB updated the literature search and participated in writing the manuscript.

### Conflict of interest statement

The authors declare that the research was conducted in the absence of any commercial or financial relationships that could be construed as a potential conflict of interest. The reviewer GT and handling editor declared their shared affiliation at time of review.

## Footnotes

^1^https://amstar.ca/docs/AMSTARguideline.pdf
