# Supplementary material for: Mapping Systematic Reviews on Forensic Psychiatric Care: A Systematic Review Identifying Knowledge Gaps
Source: Front Psychiatry. 2018 Sep 25;9:452. doi: 10.3389/fpsyt.2018.00452 (PMC6167556; doi:10.3389/fpsyt.2018.00452)
Supplement: Supplementary file 1 [file Table_1.DOCX]

**Supplementary Material I:**

**Articles excluded because high risk of bias**

| Allely, C.S. (2018). A systematic PRISMA review of individuals with autism spectrum disorder in secure psychiatric care: prevalence, treatment, risk assessment and other clinical considerations. *Journal of Criminal Psychology, 8,* 58-79. |
| --- |
| Browne, C., & Smith, I.C. (2018). Psychological interventions for anger and aggression in people with intellectual disabilities in forensic services. *Aggression and Violent Behavior,* *39* (March–April), 1-14. |
| Clarke, C., Lumbard, D., Sambrook, S., & Kerr, K. (2016). What does recovery mean to a forensic mental health patient? A systematic review and narrative synthesis of the qualitative literature. *Journal of Forensic Psychiatry & Psychology, 27*, 38-54. |
| Cohen, G., & Harvey, J. (2016). The use of psychological interventions for adult male sex offenders with a learning disability: A systematic review. *Journal of Sexual Aggression, 22*, 206-223. doi:10.1080/13552600.2015.1077279 |
| Coutinho, B. V., Hansen, A. L., Waage, L., Hillecke, T. K., & Koenig, J. (2015a). Music making interventions with adults in the forensic setting—A systematic review of the literature—Part I: Group interventions. *Music and Medicine, 7*(3), 40-53. |
| Duncan, E.A.S., Nicol, M.M., Ager, A., & Dalgleish, L. (2006). A systematic review of structured group interventions with mentally disordered offenders. *Criminal Behaviour and Mental Health, 16*(4), 217-241. |
| Fazel, S., & Seewald, K. (2012). Severe mental illness in 33,588 prisoners worldwide: systematic review and meta-regression analysis. *The British Journal of Psychiatry, 200*(5), 364-373. |
| Fielenbach, S., Donkers, F.C.L., Spree, M., Visser, H.A., & Bogaerts, S. (2018). Neurofeedback training for psychiatric disorders associated with criminal offending: A review. *Frontiers in Psychiatry*, 8, 313 |
| Golenkov, A., Nielssen, O., & Large, M. (2014). Systematic review and meta-analysis of homicide recidivism and schizophrenia. *BMC Psychiatry, 14*, 46. |
| Guy, L.S., Douglas, K.S., & Hendry, M.C. (2010). The role of psychopathic personality disorder in violence risk assessments using the HCR-20. *Journal of Personality Disorders, 24*(5), 551-580. doi:10.1521/pedi.2010.24.5.551 |
| Johnson, M. E. (2010). Violence and restraint reduction efforts on inpatient psychiatric units. *Issues in Mental Health Nursing, 31*(3), 181-197. |
| Kip, H., Bouman, Y.H.A., Kelders, S.M., & vanGemert-Pinjen, L.J.E.W.C. (2018). eHealth in treatment of offenders in forensic mental health: A review of the current state. *Frontiers in Psychiatry,* 9, 42. |
| Kivisto, A.J. (2016). Violence risk assessment and management in outpatient clinical practice. *Journal of Clinical Psychology, 72*(4), 329-349. doi:10.1002/jclp.22243 |
| Lange, S., Rehm, J., & Popova, S. (2011). The effectiveness of criminal justice diversion initiatives in North America: A systematic literature review. *International Journal of Forensic Mental Health, 10*(3), 200-214. doi:10.1080/14999013.2011.598218 |
| Lowenstein, J., Purvis, C., & Rose, K. (2016). A systematic review on the relationship between antisocial, borderline and narcissistic personality disorder diagnostic traits and risk of violence to others in a clinical and forensic sample. *Borderline Personal Disord Emot Dysregul, 3*, 14. doi:10.1186/s40479-016-0046-0 |
| Marotta, P.L. (2015). A systematic review of behavioral health interventions for sex offenders with intellectual disabilities. *Sexual Abuse*, 29(2), 148-185. doi: 10.1177/1079063215569546 |
| Marquant, T., Sabbe, B., Van Nuffel, M., & Goethals, K. (2016). Forensic assertive community treatment: A review of the literature. *Community Mental Health Journal,* 52, 873-881. |
| Mars, M., Ramlall, S., & Kaliski, S. (2012). Forensic telepsychiatry: A possible solution for South Africa? *African Journal of Psychiatry, 15*(4), 244-247. |
| Martin, M.S., Dorken, S.K., Wamboldt, A.D., & Wootten, S.E. (2012). Stopping the revolving door: A meta-analysis on the effectiveness of interventions for criminally involved individuals with major mental disorders. *Law and Human Behavior, 36*(1), 1-12. |
| Mokros, A., Vohs, K., & Habermeyer, E. (2014). Psychopathy and violent reoffending in German-speaking countries: A meta-analysis. *European Journal of Psychological Assessment, 30*(2), 117-129. doi:10.1027/1015-5759/a000178 |
| Morgan, R.D., Flora, D.B., Kroner, D.G., Mills, J.F., Varghese, F., & Steffan, J.S. (2011). Treating offenders with mental illness: A research synthesis. *Law and Human Behavior, 36*(1), 1-16. doi:10.1007/s10979-011-9271-7 |
| O’Shea, L.E., & Dickens, G.L. (2014). Short-Term Assessment of Risk and Treatability (START): Systematic review and meta-analysis. *Psychological Assessment, 26*(3), 990-1002. doi: 10.1037/a0036794 |
| Pickard, H., & Fazel, S. (2013). Substance abuse as a risk factor for violence in mental illness: Some implications for forensic psychiatric practice and clinical ethics. *Current Opinion in Psychiatry, 26*(4), 349-354. doi:10.1097/YCO.0b013e328361e798 |
| Ramesh, T., Igoumenou, A., Vazquez Montes, M., & Fazel, S. (2018). Use of risk assessment instruments to predict violence in forensic psychiatric hospitals: a systematic review and meta-analysis. *European Psychiatry,* *52*, 47–53. |
| Richards, M., Doyle, M., & Cook, P. (2009). A literature review of family interventions for dual diagnosis: Implications for forensic mental health services. *British Journal of Forensic Practice, 11*(4), 39-49. doi:10.1108/14636646200900027 |
| Robinson, J., Craig, L. ., & Tonkin, M. (2016). Perceptions of social climate and aggressive behavior in forensic services: A systematic review. *Trauma, Violence & Abuse*. doi: 10.1177/1524838016663936 |
| Ryan, S., Brown, C.K., & Watanabe-Galloway, S. (2010). Toward successful postbooking diversion: What are the next steps? *Psychiatric Services, 61*(5), 469-477. doi:10.1176/appi.ps.61.5.469 |
| Sedgwick, O., Young, S., Das, M., & Kumari, V. (2016). Objective predictors of outcome in forensic mental health services-a systematic review. *CNS Spectrums*, 1-15. doi: 10.1017/S1092852915000723 |
| Sharf, A.J., Rogers, R., Williams, M.M., & Henry, S.A. (2017). The Effectiveness of the MMPI-2-RF in detecting feigned mental disorders and cognitive deficits: A meta-analysis. *Journal of Psychopathology and Behavioral Assessment,* 39, 441-455. doi: 10.1007/s10862-017-9590-1 |
| Shepherd, A., Doyle, M., Sanders, C., & Shaw, J. (2016). Personal recovery within forensic settings – systematic review and meta-synthesis of qualitative methods studies. *Criminal Behaviour and Mental Health, 26*, 59-75. |
| Singh, J.P., & Fazel, S. (2010). Forensic risk assessment: A metareview. *Criminal Justice and Behavior, 37*(9), 965-988. doi:10.1177/0093854810374274 |
| Stewart, D., Van der Merwe, M., Bowers, L., Simpson, A., & Jones, J. (2010). A review of interventions to reduce mechanical restraint and seclusion among adult psychiatric inpatients. *Issues in Mental Health Nursing, 31*(6), 413-424. doi:10.3109/01612840903484113 |
| Sturgeon, M., Tyler, N., & Gannon, T. A. (2018). A systematic review of group work interventions in UK high secure hospitals. *Aggression and Violent Behavior,* 38, 53-75. |
| Walker, H., Tulloch, L., & Martin, C. (2012). Are they worth it? A systematic review of QOL instruments for use with mentally disordered offenders who have a diagnosis of psychosis. *British Journal of Forensic Practice, 14*(4), 252-268. doi:10.1108/14636641211283066 |

**Supplementary Material II: Literature search strategies**

PsycInfo via EBSCO 26 October 2016

Title: forensic psychiatry/mentally ill offenders

| Search terms | | Items found |
| --- | --- | --- |
| Setting: persons within forensic institutions /mentally ill offenders | | |
|  | DE "Forensic Psychiatry" OR DE "Forensic Psychology" OR DE "Mentally Ill Offenders" | 12,870 |
|  | (DE "Crime" OR DE "Criminal Behavior" OR DE "Criminal Rehabilitation" OR DE "Criminals" OR DE "Juvenile Delinquency" OR DE "Female Delinquency" OR DE "Male Delinquency" OR DE "Maximum Security Facilities" OR DE "Correctional Institutions" OR DE "Reformatories") AND (DE "Affective Disorders" OR DE "Bipolar Disorder" OR DE "Disruptive Mood Dysregulation Disorder" OR DE "Major Depression" OR DE "Mania" OR DE "Seasonal Affective Disorder" OR DE "Anxiety Disorders" OR DE "Acute Stress Disorder" OR DE "Castration Anxiety" OR DE "Death Anxiety" OR DE "Generalized Anxiety Disorder" OR DE "Obsessive Compulsive Disorder" OR DE "Panic Disorder" OR DE "Phobias" OR DE "Post-Traumatic Stress" OR DE "Posttraumatic Stress Disorder" OR DE "Separation Anxiety Disorder" OR DE "Chronic Mental Illness" OR DE "Chronic Psychosis" OR DE "Dementia" OR DE "AIDS Dementia Complex" OR DE "Dementia with Lewy Bodies" OR DE "Presenile Dementia" OR DE "Semantic Dementia" OR DE "Senile Dementia" OR DE "Vascular Dementia" OR DE "Dissociative Disorders" OR DE "Depersonalization" OR DE "Depersonalization/Derealization Disorder" OR DE "Dissociative Identity Disorder" OR DE "Fugue Reaction" OR DE "Factitious Disorders" OR DE "Munchausen Syndrome" OR DE "Hoarding Disorder" OR DE "Hoarding Behavior" OR DE "Hysteria" OR DE "Mass Hysteria" OR DE "Impulse Control Disorders" OR DE "Explosive Disorder" OR DE "Neurosis" OR DE "Childhood Neurosis" OR DE "Experimental Neurosis" OR DE "Occupational Neurosis" OR DE "Traumatic Neurosis" OR DE "Paraphilias" OR DE "Apotemnophilia" OR DE "Exhibitionism" OR DE "Fetishism" OR DE "Incest" OR DE "Pedophilia" OR DE "Sexual Masochism" OR DE "Sexual Sadism" OR DE "Transvestism" OR DE "Voyeurism" OR DE "Personality Disorders" OR DE "Antisocial Personality Disorder" OR DE "Avoidant Personality Disorder" OR DE "Borderline Personality Disorder" OR DE "Dark Triad" OR DE "Dependent Personality Disorder" OR DE "Histrionic Personality Disorder" OR DE "Narcissistic Personality Disorder" OR DE "Obsessive Compulsive Personality Disorder" OR DE "Paranoid Personality Disorder" OR DE "Passive Aggressive Personality Disorder" OR DE "Sadomasochistic Personality" OR DE "Schizoid Personality Disorder" OR DE "Schizotypal Personality Disorder" OR DE "Psychosis" OR DE "Acute Psychosis" OR DE "Affective Psychosis" OR DE "Alcoholic Psychosis" OR DE "Capgras Syndrome" OR DE "Childhood Psychosis" OR DE "Chronic Psychosis" OR DE "Experimental Psychosis" OR DE "Hallucinosis" OR DE "Paranoia (Psychosis)" OR DE "Postpartum Psychosis" OR DE "Reactive Psychosis" OR DE "Schizophrenia" OR DE "Senile Psychosis" OR DE "Toxic Psychoses" OR DE "Attention Deficit Disorder" OR DE "Attention Deficit Disorder with Hyperactivity" OR DE "Behavior Disorders" OR DE "Addiction" OR DE "Attempted Suicide" OR DE "Drug Abuse" OR DE "Homicide" OR DE "Juvenile Delinquency" OR DE "Self-Mutilation" OR DE "Substance Use Disorder" OR DE "Psychopathology" OR DE "Mental Disorders" OR DE "Adjustment Disorders" OR DE "Affective Disorders" OR DE "Alexithymia" OR DE "Anxiety Disorders" OR DE "Autism Spectrum Disorders" OR DE "Chronic Mental Illness" OR DE "Dementia" OR DE "Dissociative Disorders" OR DE "Eating Disorders" OR DE "Elective Mutism" OR DE "Factitious Disorders" OR DE "Gender Identity Disorder" OR DE "Hoarding Disorder" OR DE "Hysteria" OR DE "Impulse Control Disorders" OR DE "Koro" OR DE "Mental Disorders due to General Medical Conditions" OR DE "Neurosis" OR DE "Paraphilias" OR DE "Personality Disorders" OR DE "Pseudodementia" OR DE "Psychosis" OR DE "Schizoaffective Disorder") | 26,302 |
|  | TI(Forensic*) OR AB(forensic*) | 15,018 |
|  | TX ((Hospital) N3 (secur*)) | 976 |
|  | TX ((Maximum OR High* OR medium OR Low* OR Minimum OR forensic*) W2 (secur*)) | 3,032 |
|  | TX ((Offender* OR criminal* OR offending OR offend* OR forensic OR incarcerate* OR justice* OR delinquent* OR inmate* OR correctional OR prison* OR "violent offence*" OR reoffend* OR re-offend*) N2 (psych* OR mental* OR intellectual* OR Schizo* OR "personality disorder*" OR borderline OR antisocial OR Firesetting* OR Pyroman* OR Arson OR arsons* OR Paraphil* OR pedophil* OR paedophil* OR Hallucinat* OR "dual disord*")) | 43,736 |
|  | TX ("secure psychiatric service*" OR "secure psychiatric facilit*" OR "secure setting*" OR "secure hospital" OR "secure psychiatric" OR "secure environment*") | 797 |
|  | 1 OR 2 OR 3 OR 4 OR 5 OR 6 OR 7 | 69,521 |
| Study types: systematic reviews or meta-analysis | | |
|  | DE ("Meta Analysis" OR ZC "systematic review" OR ZC "meta analysis") OR TX ((systematic* N3 review*) OR TX (metaanaly* OR meta-analy* OR "meta analy*")) OR TX ((systematic* n3 bibliographic*) OR (systematic* n3 literature) OR (comprehensive* n3 literature) OR (comprehensive* n3 bibliographic*) OR (integrative n3 review) OR (information n2 synthesis) OR (data n2 synthesis) OR (data n2 extract*)) OR JN ("Cochrane Database of Systematic Reviews")) | 51,469 |
| Combined sets | | |
|  | 8 AND 9 | 961 |
|  | **10 AND** **Limiters - Publication Type: All Journals; Language: Danish, English, Norwegian, Swedish** | 726 |

AB = Abstract

AU = Author

DE = Term from the thesaurus

MH = Term from the “Cinahl Headings” thesaurus

MM = Major Concept

TI = Title

TX = All Text. Performs a keyword search of all the  database's searchable fields

ZC = Methodology Index

* = Truncation

“ “ = Citation Marks; searches for an exact phrase

Scopus via Elsevier 18 April 2018

Title: forensic psychiatry /mentally ill offenders

| Search terms | | Items found |
| --- | --- | --- |
| Setting: persons within forensic institutions /mentally ill offenders | | |
|  | ( KEY ("Forensic Psychiatry" OR "Commitment Of Mentally Ill" )) | 17,729 |
|  | (KEY ("Criminals" OR "Offender" OR "Prisoners" OR "Crime" OR "Prisoner" OR "Prison" OR "Criminal Behavior" OR "Prisons" )) AND (KEY ("Personality Disorder" OR "Mental Illness" OR "Psychotic Disorders" OR "Psychiatric Treatment" OR "Mentally Ill" OR "Mood Disorder" OR "Mental Disease" OR "Mental Disorders" OR "Mental Patient" OR "Mental Health Service" OR "Mental Hospital" OR "Schizophrenia" OR "Mentally Ill Persons" OR "Antisocial Personality Disorder" OR "Hospitals, Psychiatric" OR "Mental Health Care" OR "Psychopathy" )) | 18,497 |
|  | TITLE-ABS-KEY ("Forensic psychiatr*" OR "forensic institute*" OR "forensic inpatient*" OR "forensic patient*" OR "forensic out-patient*" OR "forensic outpatient*" OR "forensic clinical practice*" OR "forensic hospital*" OR "forensic treatment*" OR "forensic service*" OR "forensic ward*" OR "forensic mental" OR "forensic facili*" OR "forensic clinic*" OR "forensic neuropsych*" OR "forensic center*" OR "forensic unit*" OR "Forensic setting" OR "forensic settings" OR "forensic population" OR "forensic populations" OR "secure psychiatr*" OR "secure setting*" OR "secure hospital" OR "Maximum secur*" OR "high secur*" OR "medium secur*" OR "low* secur*" OR "minimum secur*" OR "forensic* secur*" OR "secur* forensic*") | 29,224 |
|  | 1 OR 2 OR 3 | 43,850 |
| Study types: systematic review | | |
|  | KEY("Systematic review" ) OR KEY("Meta analysis" ) OR INDEXTERMS ('systematic review' OR 'meta analysis' OR "Meta-Analysis") | 253,357 |
|  | TITLE-ABS-KEY (systematic W/2 review*) OR TITLE-ABS-KEY (meta-analy* OR metaanaly*) OR TITLE-ABS-KEY ("systematic overview*" OR "methodological overview*") | 335,614 |
|  | 5 OR 6 | 336,045 |
| Combined sets | | |
|  | 4 AND 7 | 504 |
|  | 8 AND ( LIMIT-TO ( PUBYEAR , 2018 ) OR LIMIT-TO ( PUBYEAR , 2017 ) OR LIMIT-TO ( PUBYEAR , 2016 ) | 119 |

**TITLE-ABS-KEY** = Title or abstract or keywords
**ALL =** All fields
**PRE/n =** "precedes by". The first term in the search must precede the second by a specified number of terms (n).

**W/n =** "within". The terms in the search must be within a specified number of terms (n) in any order.
***** = Truncation

**“ “** = Citation Marks; searches for an exact phrase

LIMIT-TO ( SRCTYPE , "j" = Limit to source type journal
LIMIT-TO ( DOCTYPE , "ar" = Limit to document type article
LIMIT-TO ( DOCTYPE , "re" = Limit to document type review

Scopus via Elsevier 27 October 2016

Title: forensic psychiatry /mentally ill offenders

| Search terms | | Items found |
| --- | --- | --- |
| Setting: persons within forensic institutions /mentally ill offenders | | |
|  | ( KEY ("Forensic Psychiatry" OR "Commitment Of Mentally Ill" )) | 17,540 |
|  | (KEY ("Criminals" OR "Offender" OR "Prisoners" OR "Crime" OR "Prisoner" OR "Prison" OR "Criminal Behavior" OR "Prisons" )) AND (KEY ("Personality Disorder" OR "Mental Illness" OR "Psychotic Disorders" OR "Psychiatric Treatment" OR "Mentally Ill" OR "Mood Disorder" OR "Mental Disease" OR "Mental Disorders" OR "Mental Patient" OR "Mental Health Service" OR "Mental Hospital" OR "Schizophrenia" OR "Mentally Ill Persons" OR "Antisocial Personality Disorder" OR "Hospitals, Psychiatric" OR "Mental Health Care" OR "Psychopathy" )) | 17,408 |
|  | TITLE-ABS-KEY ("Forensic psychiatr*" OR "forensic institute*" OR "forensic inpatient*" OR "forensic patient*" OR "forensic out-patient*" OR "forensic outpatient*" OR "forensic clinical practice*" OR "forensic hospital*" OR "forensic treatment*" OR "forensic service*" OR "forensic ward*" OR "forensic mental" OR "forensic facili*" OR "forensic clinic*" OR "forensic neuropsych*" OR "forensic center*" OR "forensic unit*" OR "Forensic setting" OR "forensic settings" OR "forensic population" OR "forensic populations" OR "secure psychiatr*" OR "secure setting*" OR "secure hospital" OR "Maximum secur*" OR "high secur*" OR "medium secur*" OR "low* secur*" OR "minimum secur*" OR "forensic* secur*" OR "secur* forensic*") | 26,649 |
|  | 1 OR 2 OR 3 | 40,618 |
| Study types: systematic review | | |
|  | KEY("Systematic review" ) OR KEY("Meta analysis" ) OR INDEXTERMS ('systematic review' OR 'meta analysis' OR "Meta-Analysis") | 194,108 |
|  | TITLE-ABS-KEY (systematic W/2 review*) OR TITLE-ABS-KEY (meta-analy* OR metaanaly*) OR TITLE-ABS-KEY ("systematic overview*" OR "methodological overview*") | 268,911 |
|  | 5 OR 6 | 269,276 |
| Combined sets | | |
|  | 4 AND 7 | 397 |
|  | 8 AND ( LIMIT-TO ( LANGUAGE , "English" ) OR LIMIT-TO ( LANGUAGE , "Swedish" ) ) | 371 |

**TITLE-ABS-KEY** = Title or abstract or keywords
**ALL =** All fields
**PRE/n =** "precedes by". The first term in the search must precede the second by a specified number of terms (n).

**W/n =** "within". The terms in the search must be within a specified number of terms (n) in any order.
***** = Truncation

**“ “** = Citation Marks; searches for an exact phrase

LIMIT-TO ( SRCTYPE , "j" = Limit to source type journal
LIMIT-TO ( DOCTYPE , "ar" = Limit to document type article
LIMIT-TO ( DOCTYPE , "re" = Limit to document type review

Campbell Library 27 October 2016

Title: forensic psychiatry/mentally ill offenders

| Search terms | | Items found |
| --- | --- | --- |
| Setting: persons within forensic institutions / mentally ill offenders | | |
|  | Forensic | 3 |
|  | ((offender* OR criminal* OR offending OR offend* OR forensic OR incarcerate* OR justice* OR delinquent* OR inmate* OR correctional OR prison* OR "violent offence*" OR reoffend* OR re-offend*) AND (psych* OR mental* OR intellectual* OR Schizo* OR disorder* OR borderline OR antisocial OR Firesetting* OR Pyroman* OR Arson OR arsons* OR Paraphil* OR pedophil* OR paedophil* OR Hallucinat*)) | 0 |
|  | "Mentally ill offender*" | 0 |
|  | Mentally ill offender | 1 |
|  | Secure hospital | 1 |

Cochrane Library via Wiley 27 October 2016 (CDSR, DARE & HTA)

Title: forensic psychiatry/ mentally ill offenders

| Search terms | | Items found |
| --- | --- | --- |
| Setting: persons within forensic institutions / mentally ill offenders | | |
|  | MeSH descriptor: [Forensic Psychiatry] | 202 |
|  | ([mh Prisoners] OR [mh Crime] OR [mh Criminals] OR [mh "Juvenile Delinquency"] OR [mh "Residential Treatment"] OR [mh Prisons]) AND ([mh "Mental Disorders"] OR [mh "Hospitals, Psychiatric"] OR [mh "Mentally ill Persons"] OR [mh "Psychoses, Substance-Induced"] OR [mh "Psychiatry"] OR [mh "Diagnosis, Dual (Psychiatry)"] OR [mh "Schizophrenic Psychology"] OR [mh "Delusions"]) | 1157 |
|  | (Offender* or criminal* or offending or offend* or forensic or incarcerate* or justice* or delinquent* or inmate* or correctional or prison* or "violent offence*" or reoffend* or re-offend*) and (psych* or mental* or intellectual* or Schizo* or disorder* or borderline or antisocial or Firesetting* or Pyroman* or Arson or arsons* or Paraphil* or pedophil* or paedophil* or Hallucinat*):ti,ab,kw | 1147 |
|  | (Forensic* NEAR/2 (psychiatr* OR institution* OR inpatient* OR patient* OR out-patient* OR outpatient* OR "clinical practice" OR hospital* OR treatment* OR service* OR ward* OR mental* OR facilit* OR clinic* OR neuropsych* OR "personality disorder" OR Center* OR unit*)):ti,ab,kw | 129 |
|  | ("Forensic setting" or "forensic settings" or "forensic population" or "forensic populations "):ti,ab,kw | 17 |
|  | ((Hospital OR hospitals) NEAR/3 ("maximum secur*" OR "high secur*" OR "high secur*" OR medium OR "low secur*" OR "lower secur*" OR minimum*)):ti,ab,kw | 59 |
|  | ((Maximum OR high* OR medium OR low* OR minimum OR forensic*) NEXT/1 (secur*)) | 57 |
|  | (Offender* OR criminal* OR offending OR offend OR forensic OR incarcerate* OR "juvenile justice system*" OR delinquent* OR inmate* OR "correctional population*" OR "correctional setting*" OR "correctional facilit*" OR "correctional institution*" OR "correctional mental health" OR prison* OR "violent offence*" OR reoffend* OR re-offend*) NEAR/4 ("mentally disorder*" OR "mental disorder" OR "mental disabilit*" OR "mental health" OR "mentally ill" OR "mental illness" OR "personality disorder*" OR "intellectual disability" OR "intellectually disabled" OR psychiat* OR psychot* OR psychosis OR schizo* OR borderline OR "paranoid disorder*" OR antisocial OR "high risk" OR "dual disorder*" OR Hallucinat* OR delusions OR delusional) | 326 |
|  | ("secure psychiatric service*" OR "secure psychiatric facilit*" OR "secure setting*" OR "secure hospital" OR "secure psychiatric" OR "secure environment*"):ti,ab,kw | 21 |
|  | 1 OR 2 OR 3 OR 4 OR 5 OR 6 OR 7 OR 8 OR 9 | CDSR/57DARE/83HTA/27 |

[AU] = Author

[MAJR] = MeSH Major Topic

[MeSH] = Term from the Medline controlled vocabulary, including terms found below this term in the MeSH hierarchy

[MeSH:NoExp] = Does not include terms found below this term in the MeSH hierarchy

Systematic[SB] = Filter for retrieving systematic reviews

[TI] = Title

[TIAB] = Title or abstract

[TW] = Text Word

* = Truncation

“ “ = Citation Marks; searches for an exact phrase

CDSR = Cochrane Database of Systematic Review

CENTRAL = Cochrane Central Register of Controlled Trials, “trials”

CRM = Method Studies

DARE = Database Abstracts of Reviews of Effects, “other reviews”

EED = Economic Evaluations

HTA = Health Technology Assessments

PubMed via NLM 28 October 2016

Title: forensic psychiatry/ mentally ill offenders (complimentary search to find non-indexed references)

| Search terms | | Items found |
| --- | --- | --- |
| Setting: persons within forensic institutions /mentally ill offenders | | |
|  | ("Forensic psychiatr*"[tiab] OR "forensic institute*"[tiab] OR "forensic inpatient*"[tiab] OR "forensic patient*"[tiab] OR "forensic out-patient*"[tiab] OR "forensic outpatient*"[tiab] OR "forensic clinical practice*"[tiab] OR "forensic hospital*"[tiab] OR "forensic treatment*"[tiab] OR "forensic service*"[tiab] OR "forensic ward*"[tiab] OR "forensic mental"[tiab] OR "forensic facili*"[tiab] OR "forensic clinic*"[tiab] OR "forensic neuropsych*"[tiab] OR "forensic center*"[tiab] OR "forensic unit*"[tiab] OR "Forensic setting"[tiab] OR "forensic settings"[tiab] OR "forensic population"[tiab] OR "forensic populations"[tiab] OR "secure psychiatr*"[tiab] OR "secure setting*"[tiab] OR "secure hospital"[tiab] OR "Maximum secur*"[tiab] OR "high secur*"[tiab] OR "medium secur*"[tiab] OR "low* secur*"[tiab] OR "minimum secur*"[tiab] OR "forensic* secur*"[tiab] OR "secur* forensic*"[tiab]) NOT Medline[SB] | 159 |
|  | ((Offender*[tiab] OR criminal*[tiab]OR offending[tiab] OR offend*[tiab] OR forensic[tiab] OR incarcerate*[tiab] OR justice*[tiab] OR delinquent*[tiab] OR inmate*[tiab] OR correctional[tiab] OR prison*[tiab] OR "violent offence*"[tiab] OR reoffend*[tiab] OR re-offend*[tiab]) AND (psychiatri*[tiab] OR psycholog[tiab] OR mental*[tiab] OR intellectual*[tiab] OR Schizo*[tiab] OR "personality disorder*"[tiab] OR borderline[tiab] OR antisocial[tiab] OR Firesetting*[tiab] OR Pyroman*[tiab] OR arson*[tiab] OR Paraphil*[tiab] OR pedophil*[tiab] OR paedophil*[tiab] OR Hallucinat*[tiab] OR "dual disord*"[tiab])) NOT medline[SB] | 1714 |
|  | 1 OR 2 | 1791 |
| Combined sets, limited to study type: systematic review | | |
|  | 3 AND Systematic[SB] | 68 |

[MeSH] = Term from the Medline controlled vocabulary, including terms found below this term in the MeSH hierarchy

[MeSH:NoExp] = Does not include terms found below this term in the MeSH hierarchy

[MAJR] = MeSH Major Topic

[TIAB] = Title or abstract

[TI] = Title

[AU] = Author

[TW] = Text Word

Systematic[SB] = Filter for retrieving systematic reviews

* = Truncation

SocIndex via EBSCO 27 October 2016

Title: forensic psychiatry/ mentally ill offenders

| Search terms | | Items found |
| --- | --- | --- |
| Setting: persons within forensic institutions /mentally ill offenders | | |
|  | (DE "FORENSIC psychiatry" OR DE "MENTALLY ill criminals" OR DE "CRIMINAL psychology" OR DE "OFFENDERS with mental disabilities") | 2,491 |
|  | (DE "CRIME" OR DE "CRIMINALS" OR DE "WOMEN criminals" OR DE "JUVENILE corrections" OR DE "CORRECTIONS (Criminal justice administration)" OR DE "JUVENILE detention" OR DE "IMPRISONMENT" OR DE "INSTITUTIONALIZED persons" OR DE "PRISON population" OR DE "PRISONERS") AND (DE "PSYCHIATRIC hospitals" OR DE "MENTALLY ill" OR DE "PSYCHIATRIC diagnosis" OR DE "MENTAL illness" OR DE "PATHOLOGICAL psychology" OR DE "AFFECTIVE disorders" OR DE "BIPOLAR disorder" OR DE "COMPULSIVE behavior" OR DE "CONDUCT disorders in adolescence" OR DE "DEPERSONALIZATION" OR DE "MENTAL disabilities" OR DE "MENTAL illness" OR DE "PERSONALITY disorders" OR DE "ANTISOCIAL personality disorders" OR DE "BORDERLINE personality disorder" OR DE "IMPULSIVE personality" OR DE "MASOCHISM" OR DE "NARCISSISTIC personality disorder" OR DE "OBSESSIVE-compulsive personality disorder" OR DE "PSYCHOSEXUAL disorders" OR DE "SADISM" OR DE "SUBSTANCE abuse" OR DE "PSYCHOSES" OR DE "PSYCHOSEXUAL disorders" OR DE "PEDOPHILIA" OR DE "Schizophrenics" OR DE "PSYCHOPATHY") | 1,357 |
|  | TI(Forensic*) OR AB(forensic*) | 6,582 |
|  | TX ((Hospital) N3 (secur*)) | 1,158 |
|  | TX ((Maximum OR High* OR medium OR Low* OR Minimum OR forensic*) W2 (secur*)) | 8,472 |
|  | TX ((Offender* OR criminal* OR offending OR offend* OR forensic OR incarcerate* OR justice* OR delinquent* OR inmate* OR correctional OR prison* OR "violent offence*" OR reoffend* OR re-offend*) N2 (psych* OR mental* OR intellectual* OR Schizo* OR "personality disorder*" OR borderline OR antisocial OR Firesetting* OR Pyroman* OR Arson OR arsons* OR Paraphil* OR pedophil* OR paedophil* OR Hallucinat* OR "dual disord*")) | 27,781 |
|  | TX ("secure psychiatric service*" OR "secure psychiatric facilit*" OR "secure setting*" OR "secure hospital" OR "secure psychiatric" OR "secure environment*") | 1,551 |
|  | 1 OR 2 OR 3 OR 4 OR 5 OR 6 OR 7 OR 8 | 41,158 |
| Study types: systematic review | | |
|  | DE "Systematic reviews (Medical research)" OR DE "Meta-analysis" | 3,240 |
|  | (TI (systematic* n3 review*)) or (AB (systematic* n3 review*)) or (TI (systematic* n3 bibliographic*)) or (AB (systematic* n3 bibliographic*)) or (TI (systematic* n3 literature)) or (AB (systematic* n3 literature)) or (TI (comprehensive* n3 literature)) or (AB (comprehensive* n3 literature)) or (TI (comprehensive* n3 bibliographic*)) or (AB (comprehensive* n3 bibliographic*)) or (TI (integrative n3 review)) or (AB (integrative n3 review)) or (JN "Cochrane Database of Systematic Reviews") or (TI (information n2 synthesis)) or (TI (data n2 synthesis)) or (AB (information n2 synthesis)) or (AB (data n2 synthesis)) or (TI (data n2 extract*)) or (AB (data n2 extract*)) or (TI (medline or pubmed or psyclit or cinahl or (psycinfo not "psycinfo database") or "web of science" or scopus or embase)) or (AB (medline or pubmed or psyclit or cinahl or (psycinfo not "psycinfo database") or "web of science" or scopus or embase)) or (TI (meta-analy* or metaanaly*)) or (AB (meta-analy* or metaanaly*)) | 7,672 |
|  | 9 OR 10 | 8,275 |
| Combined sets | | |
|  | 8 AND 11 | 371 |
|  | 12 AND Limiters - Scholarly (Peer Reviewed) Journals; Document Type: Article; Language: Danish, English, Norwegian, Swedish | 313 |

AB = Abstract

AU = Author

DE = Term from the thesaurus

MH = Term from the “Cinahl Headings” thesaurus

MM = Major Concept

TI = Title

TX = All Text. Performs a keyword search of all the  database's searchable fields

ZC = Methodology Index

* = Truncation, “ “ = Citation Marks; searches for an exact phrase

Cinahl via EBSCO 26 October 2016

Title: forensic psychiatry/mentally ill offenders

| Search terms | | Items found |
| --- | --- | --- |
| Setting: persons within forensic institutions / mentally ill offenders | | |
|  | (MH "Forensic Psychiatry+") OR (MH "Mentally Ill Offenders") | 1,915 |
|  | (MH "Prisoners" OR MH "Correctional Facilities" OR MH "Crime" OR MH "Sex Offenders") OR MH "Repeat Offenders" OR MH "Juvenile Offenders" OR MH "Juvenile Delinquency") AND (MH "Mental Disorders+" OR MH "Psychopathology" OR MH "Psychiatric Care" OR MH "Psychiatric Patients" OR MH "Mental Health Services" OR MH "Hospitals, Psychiatric") | 13,190 |
|  | TI(Forensic*) OR AB(forensic*) | 3,066 |
|  | TX ((Hospital) N3 (secur*)) | 2,258 |
|  | TX ((Maximum OR High* OR medium OR Low* OR Minimum OR forensic*) W2 (secur*)) | 2,657 |
|  | TX ((Offender* OR criminal* OR offending OR offend* OR forensic OR incarcerate* OR justice* OR delinquent* OR inmate* OR correctional OR prison* OR "violent offence*" OR reoffend* OR re-offend*) N2 (psych* OR mental* OR intellectual* OR Schizo* OR "personality disorder*" OR borderline OR antisocial OR firesetting* OR pyroman* OR arson OR arsons* OR paraphil* OR pedophil* OR paedophil* OR hallucinat* OR "dual disord*")) | 9,776 |
|  | TX ("secure psychiatric service*" OR "secure psychiatric facilit*" OR "secure setting*" OR "secure hospital" OR "secure psychiatric" OR "secure environment*") | 1,648 |
|  | 1 OR 2 OR 3 OR 4 OR 5 OR 6 OR 7 | 25,986 |
| Study types: systematic review | | |
|  | MH "Systematic Review" OR ZT "systematic review" OR MH "Meta Analysis" OR ZT "meta analysis" | 53,852 |
|  | (TI (systematic* n3 review*)) or (AB (systematic* n3 review*)) or (TI (systematic* n3 bibliographic*)) or (AB (systematic* n3 bibliographic*)) or (TI (systematic* n3 literature)) or (AB (systematic* n3 literature)) or (TI (comprehensive* n3 literature)) or (AB (comprehensive* n3 literature)) or (TI (comprehensive* n3 bibliographic*)) or (AB (comprehensive* n3 bibliographic*)) or (TI (integrative n3 review)) or (AB (integrative n3 review)) or (JN “Cochrane Database of Systematic Reviews”) or (TI (information n2 synthesis)) or (TI (data n2 synthesis)) or (AB (information n2 synthesis)) or (AB (data n2 synthesis)) or (TI (data n2 extract*)) or (AB (data n2 extract*)) or (TI (medline or pubmed or psyclit or cinahl or (psycinfo not “psycinfo database”) or “web of science” or scopus or embase)) or (AB (medline or pubmed or psyclit or cinahl or (psycinfo not “psycinfo database”) or “web of science” or scopus or embase)) or (TI (meta-analy* or metaanaly*)) or (AB (meta-analy* or metaanaly*)) | 61,746 |
|  | 9 OR 10 | 79,354 |
| Combined sets | | |
|  | 8 AND 11 | 495 |
|  | 12 AND Limiters - Publication Type: Journal Article; Language: Danish, English, Norwegian, Swedish | 467 |

AB = Abstract

AU = Author

DE = Term from the thesaurus

MH = Term from the “Cinahl Headings” thesaurus

MM = Major Concept

TI = Title

TX = All Text. Performs a keyword search of all the  database's searchable fields

ZC = Methodology Index

* = Truncation

“ “ = Citation Marks; searches for an exact phrase

Joanna Briggs Institute database via OvidSP 27 October 2016

Title: forensic psychiatry/mentally ill offenders

| Search terms | | Items found |
| --- | --- | --- |
| Setting: persons within forensic institutions / mentally ill offenders | | |
|  | (forensic* or offender* or criminal* or offending or offend* or forensic or incarcerate* or justice* or delinquent* or inmate* or correctional or prison* or "violent offence*" or reoffend* or re-offend*).mp. | 105 |
|  | limit 1 to (recommended practices or "systematic review protocols" or systematic reviews) | 70 |

.ab. =Abstract

.ab,ti. = Abstract or title

.af.= All fields

Exp= Term from the Medline controlled vocabulary, including terms found below this term in the MeSH hierarchy

.sh.= Term from the Medline controlled vocabulary

.ti. = Title

/ = Term from the Medline controlled vocabulary, but does not include terms found below this term in the MeSH hierarchy

* = Focus (if found in front of a MeSH-term)

* or $= Truncation (if found at the end of a free text term)

.mp=text, heading word, subject area node, title
